# PPGLomics: an interactive platform for pheochromocytoma and paraganglioma transcriptomics

**DOI:** 10.1530/ERC-26-0140

**Published:** 2026-09-22

**Authors:** Hussam Alkaissi, Catherine M Gordon, Karel Pacak

**Affiliations:** ^1^ *Eunice Kennedy Shriver* National Institute of Child Health and Human Development, National Institutes of Health, Bethesda, Maryland, USA; ^2^ Center of Adrenal Endocrine Tumors, AKESO, Prague 5, Czech Republic; ^3^ Faculty of Medicine, Palacky University, Olomouc, Czech Republic; ^4^ Faculty of Medicine, 5th Department of Medicine, Comenius University, Bratislava, Slovakia

**Keywords:** pheochromocytoma, paraganglioma, multiomics, bioinformatics

## Abstract

Pheochromocytomas and paragangliomas (PPGLs) are rare neuroendocrine tumors with unique biological behavior and remarkably high heritability, yet dedicated bioinformatics resources for these diagnoses remain limited. Existing cancer multi-omics platforms are pan-cancer in scope, often lacking the disease-specific annotations, granularity, and cross-database harmonization required for meaningful stratification and hypothesis generation. Here, we introduce PPGLomics, an interactive Web-based platform designed for comprehensive PPGL transcriptomics analysis. PPGLomics v1.0 integrates two major datasets, the TCGA-PCPG cohort (*n* = 160) spanning multiple molecular subtypes and the A5 Consortium SDHB cohort (*n* = 91) with detailed clinicopathological and molecular annotations. The platform provides basic and clinical scientists, as well as a broad range of healthcare professionals, with tools for differential expression analysis, correlation analysis, survival analysis, and visualization, including boxplots, heatmaps, volcano plots, and Kaplan–Meier survival plots, enabling exploration of gene expression patterns across PPGL subtypes without requiring bioinformatics expertise. PPGLomics v1.0 is freely available at https://alkaissilab.shinyapps.io/PPGLomics.

## Introduction

The publication of the human genome at the turn of the century required a substantial financial investment, international collaboration, and coordination between public and private sectors (1). In the following 25 years, sequencing costs have decreased by several orders of magnitude while throughput and accuracy have dramatically improved (2). The speed at which results are available has also dramatically increased. Whole-genome sequencing can now be performed and interpreted within a single day, shifting the primary bottleneck in genomics from data acquisition to data analysis and interpretation, all tightly linked to the individual patient scenario (2, 3). Large-scale cancer genomics initiatives, most notably The Cancer Genome Atlas (TCGA), have generated multi-omics data across different cancer types, creating unprecedented opportunities for hypothesis generation and discoveries (4). To make these data accessible to biologists and clinicians, several user-friendly Web portals have been developed by large bioinformatics teams, such as cBioPortal, GEPIA, UALCAN, OncoDB, and UCSC Xena (5, 6, 7, 8, 9, 10). These tools have democratized access to cancer genomics data and facilitated cross-cancer multi-omics comparisons, enabling researchers and clinicians without bioinformatics expertise to conduct sophisticated genomic analyses. GEPIA alone processed over 400,000 analysis requests within two years of its launch (6).

Existing platforms are pan-cancer by design and are of limited utility in rare cancer research that requires disease-specific context and annotations. Pheochromocytomas and paragangliomas (PPGLs) exemplify this challenge. Paragangliomas are tumors of autonomic ganglia (sympathetic or parasympathetic), many of which are hormonally active, secreting large amounts of catecholamines. Anatomic locations of paragangliomas infer some of their behavior. For example, head and neck paragangliomas are of parasympathetic origin, are less likely to metastasize, and more likely to follow an indolent course, while abdominal paragangliomas are of sympathetic origin with higher rates of metastasis and aggressive behavior (11, 12). These neuroendocrine tumors differ fundamentally from most malignancies, as they are slow growing yet can metastasize, often many years after the initial diagnosis (13). There is no definitive histopathological distinction between benign and malignant tumors, and virtually all tumors carry metastatic potential (14). Furthermore, PPGLs are among the most heritable cancers, with germline pathogenic variants identified in over 30% of patients and somatic drivers in approximately 40% (15, 16, 17, 18).

The biological and clinical heterogeneity of PPGLs is currently largely determined by genotype. For example, tumors driven by mutations in genes encoding enzymes of Krebs cycle (e.g. succinate dehydrogenase subunit B, *SDHB*), extra-adrenal location, larger size, and tumor-harboring *ATRX/TERT* alterations exhibit higher metastatic rates (13, 19). Meaningful PPGL transcriptomics analysis therefore requires genotype-specific stratification and disease-relevant annotations, including tumor location, size, and ATRX/TERT status, that are usually unavailable in existing pan-cancer portals. An additional challenge is the scarcity of large cohorts in rare cancer research. The two largest publicly available PPGL datasets are the TCGA-PCPG cohort (*n* = 179, 19 excluded after pan-cancer quality control harmonization), spanning multiple genotypes with both primary and metastatic tumors, and the A5 Consortium cohort (*n* = 91), comprising *SDHB*-driven tumors across anatomic locations with primary and metastatic tumors (16, 19).

To address this gap, we have developed PPGLomics v1.0, a free interactive Web-based platform dedicated to PPGL transcriptomics analysis, with disease-specific annotations, providing a specialized tool for hypothesis generation and data exploration (Link: https://alkaissilab.shinyapps.io/PPGLomics).

## Methods

### Data sources and processing

PPGLomics v1.0 integrates two publicly available PPGL transcriptomic datasets, the TCGA-PCPG and the A5 Consortium (16). RNA sequencing data for both cohorts were downloaded from cBioPortal; clinical and molecular annotations were obtained from the original publications.

The TCGA-PCPG dataset comprises 160 pheochromocytoma and paraganglioma samples (of 179 original cases following pan-cancer quality control) spanning multiple driver genotypes, including *SDHx, VHL, RET, EPAS1, EGLN1, HRAS, NF1, MAX, TMEM127, MAML3* fusions, *CSDE1*, and sporadic cases. Gene expression values are provided as log_2_-transformed transcripts per million (TPM). Metadata annotations include driver genotype and RNA expression cluster (pseudohypoxia, kinase signaling, Wnt-altered, and cortical admixture), lesion type (primary tumor; metastatic tumor), overall survival, and progression-free survival, as reported by Fishbein *et al*. (16). Expression data were filtered to the 15,000 most variable genes to optimize Web application performance, excluding genes with very low expression (genes with maximum expression <2 log_2_ TPM across samples were excluded).

The A5 Consortium dataset consists of 91 PPGL samples (of 94 original cases following pan-cancer quality control), all harboring germline *SDHB* pathogenic variants (truncating, missense, frameshift, deletions, or intronic variants), as reported by Flynn *et al*. Annotations include tumor anatomical location, lesion type (primary tumor, metastatic tumor, primary tumor from patients who subsequently developed metastases, and paired primary–metastatic cases), biochemical phenotype, tumor size, mutation type and position, *ATRX/TERT* alteration status, chromothripsis events, overall survival, and metastasis-free survival. The initial dataset of over 42,000 genes was filtered to remove genes with very low expression (maximum expression <2 log_2_ c.p.m.). We included protein-coding genes, as well as differentially expressed novel loci (e.g. metastatic vs primary tumors), including microRNAs and lncRNAs, to facilitate hypothesis generation and identification of new targets in PPGL biology. This filtering removed 43% of the initial expression matrix, yielding a final curated dataset of 23,928 genes. The summary of the design is shown in Fig. 1. Eight publicly available pheochromocytoma/paraganglioma microarray series (GSE19422 (20), GSE19987 (21), GSE2841 (22), GSE39716, GSE50442 (23), GSE51081 (24), GSE67066 (25), and E-MTAB-733 (26)) were assembled into a single harmonized expression compendium. Probe-level intensities were collapsed to HUGO Gene Nomenclature Committee (HGNC) gene symbols using the highest mean probe per symbol, and the analysis was restricted to the 11,374 genes measured across all platforms, yielding a matrix of 606 tumor samples. For cross-study analyses, per-sample distributions were quantile-normalized to a common reference and study-of-origin batch effects removed. For the COMETE (E-MTAB-733) series, CEL files were RMA-normalized and only the publicly annotated variables (sex, germline genotype, and molecular cluster) were included in this version. OncoPrint of the harmonized datasets is shown in Supplementary Fig. 1 (see section on Supplementary materials given at the end of the article).

**Figure 1:**
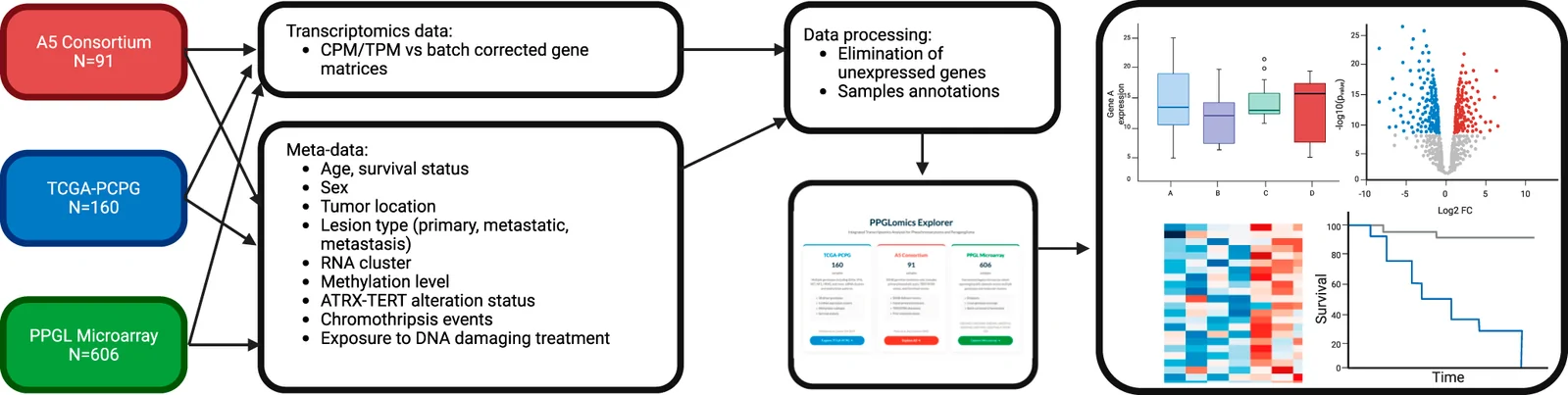
Design of the PPGLomics platform. Expression matrices for two independent publicly available PPGL cohorts, the TCGA-PCPG (*n* = 160) and the A5 Consortium (*n* = 91), and 8 microarray datasets containing 606 samples were downloaded from cBioPortal, Gene Expression Omnibus, and ArrayExpress. For each case, sample-level annotations were curated from the corresponding primary publications, including age, survival status, sex, tumor location, lesion type (metastatic; non-metastatic), molecular cluster, and other variables. Expression data were processed by eliminating unexpressed genes and integrating sample annotations. The data underlie an interactive analysis suite supporting single-gene expression, gene–gene correlation, volcano plot (differential expression), heatmap, and OncoPrint, with comparisons organized around annotations relevant to PPGL biology, such as genotype, molecular cluster, biochemical phenotype, and metastatic status.

### Web application implementation

PPGLomics was developed as an interactive Web application using the R Shiny package. The application implements i) single-gene expression analysis with boxplots and Kruskal–Wallis testing for nonparametric multi-group comparisons; ii) hierarchical clustering heatmaps with Z-score normalization; iii) pairwise gene correlation analysis using Spearman or Pearson correlation methods; iv) genome-wide similar gene identification via vectorized correlation; v) differential expression analysis using Wilcoxon rank-sum tests with Benjamini–Hochberg correction, visualized as interactive volcano plots; vi) Kaplan–Meier survival analysis with log-rank testing; and vii) OncoPrint visualization of driver alterations. An example is shown in Fig. 2.

**Figure 2:**
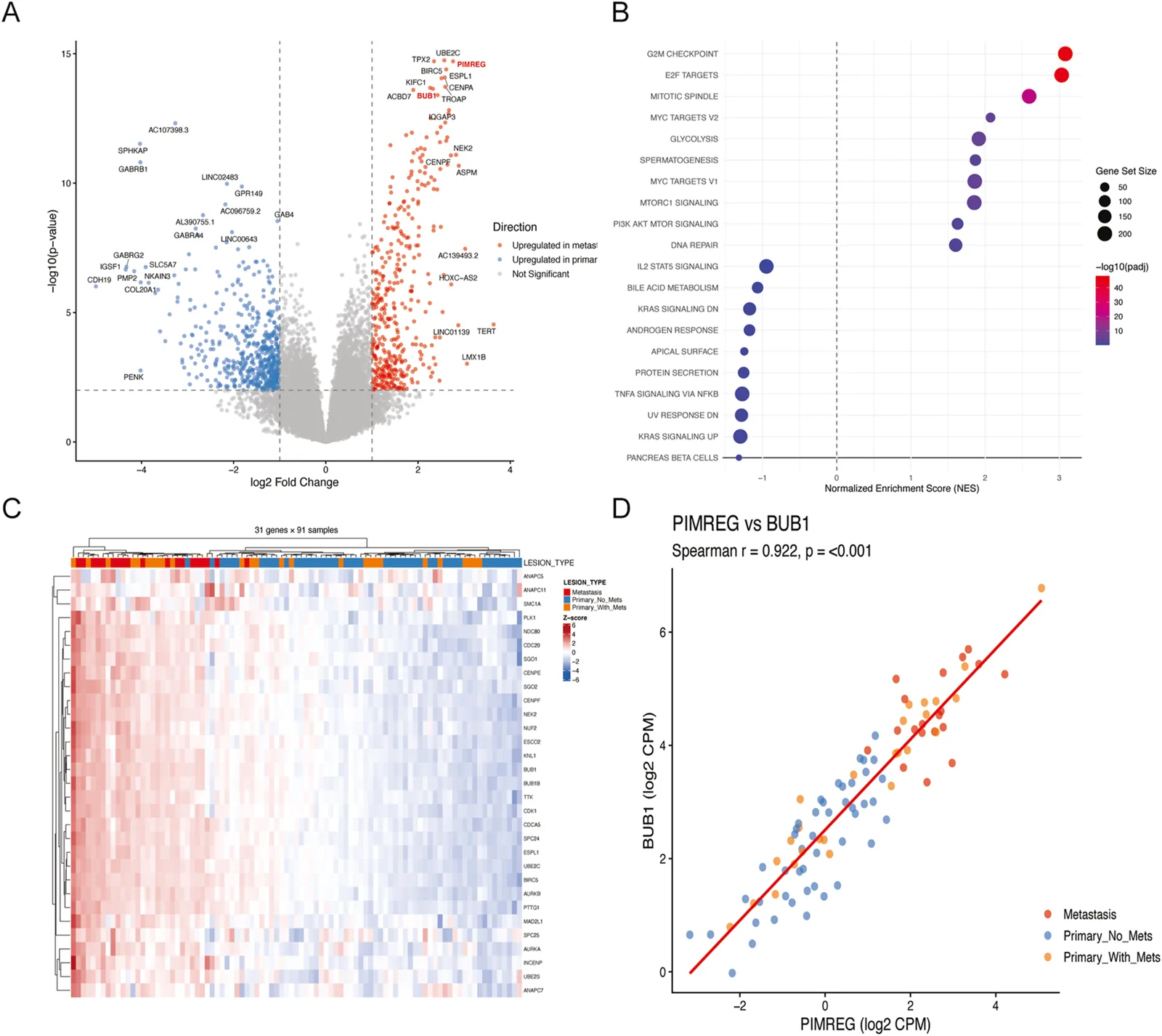
Mitotic spindle gene expression signature distinguishes metastatic *SDHB* PPGLs from primary *SDHB* PPGLs. (A) Volcano plot of differentially expressed genes between metastatic (*n* = 19) and non-metastatic primary PPGLs (*n* = 46). The red and blue points indicate genes significantly upregulated in metastases vs non-metastatic primary tumors, respectively. The dashed lines denote significance thresholds (adjusted *P* < 0.05, log_2_ fold change >1). (B) Gene set enrichment analysis (GSEA) demonstrating enrichment of cell cycle and mitotic pathways in metastatic samples. (C) Heatmap of 31 selected mitotic spindle-related genes across 91 samples. Column annotations indicate lesion type: non-metastatic primary (blue), primary with subsequent metastasis (yellow), and metastasis (red). Expression values are z-score normalized. (D) Correlation between *PIMREG* and *BUB1* expression (Spearman *R* = 0.92, *P* < 0.001).

Core R packages include Shiny for the Web framework, ggplot2 and plotly for static and interactive visualizations, ComplexHeatmap for heatmaps and mutation landscapes, survival and survminer for Kaplan–Meier analysis, and matrixTests for vectorized statistical testing. The application was developed with AI assistance (Anthropic Claude) for code generation, with iterative testing and domain-specific refinement by the investigator. Network analysis was performed by downloading the top 25 genes correlated with BUB1 from PPGLomics and analyzing them in the network analysis software Cytoscape (version 3.10.4). Gene set enrichment analysis (GSEA) was performed in R.

### Case study: mitotic spindle gene expression signature in metastatic PPGLs

To identify transcriptomic signatures associated with metastatic progression in *SDHB*-driven PPGLs, we performed differential gene expression analysis comparing primary tumors without metastasis (*n* = 46) to metastatic lesions (*n* = 19) using the A5 Consortium dataset. This analysis revealed striking enrichment of genes involved in cell cycle regulation (E2F targets; G2M checkpoint), mitotic spindle assembly, and chromosome segregation among the most significantly upregulated transcripts in metastatic *SDHB* PPGLs (Fig. 2A and B). A focused analysis of 31 mitotic spindle-related genes demonstrated coordinated upregulation in metastatic samples, with notably elevated expression also observed in a subset of primary *SDHB* tumors that subsequently developed metastases (Fig. 2C). Among these genes, *PIMREG* (PICALM interacting mitotic regulator) and *BUB1* (budding uninhibited by benzimidazoles 1) emerged as potential markers of metastatic potential. Expression of these two genes was strongly correlated (Spearman *R* = 0.92, *P* < 0.001; Fig. 2D), suggesting coordinated activation of the mitotic program.

Both *PIMREG* and *BUB1* displayed stepwise expression across disease states, with lowest expression in primary tumors without metastasis (median log_2_ c.p.m. = −0.21 and 2.35, respectively), intermediate in primary tumors that later metastasized (median = 1.11 and 3.38, respectively), and highest in metastatic lesions (median = 2.57 and 4.37; Kruskal–Wallis *P* = 5.87 × 10^−9^ and 4.94 × 10^−9^, respectively, Fig. 3A and B). Analysis of paired primary and metastatic *SDHB* samples from the same patients confirmed this pattern, with *PIMREG* and *BUB1* consistently upregulated upon metastatic progression in the majority of cases (Fig. 3E and F). Notably, expression of both genes was higher in tumors from patients previously treated with DNA-damaging agents, such as CVD chemotherapy or ^131^I-MIBG (Fig. 3C and D), consistent with prior observations that alkylating agent exposure increases tumor mutational burden and may select aggressive clones (27). Importantly, high *PIMREG* and *BUB1* expression in PPGLs was associated with significantly reduced metastasis-free survival (Fig. 3G and H).

**Figure 3:**
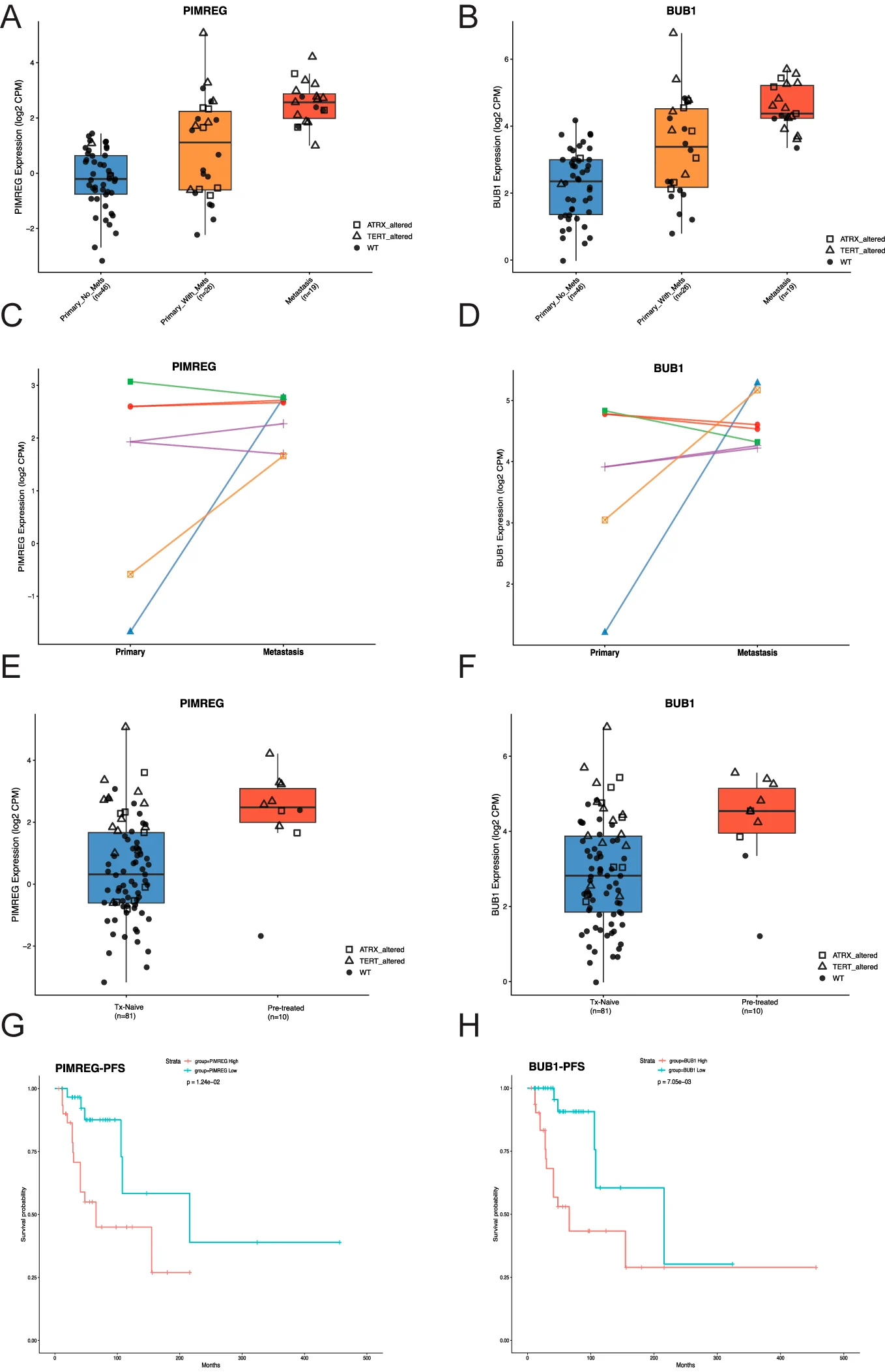
PIMREG and BUB1 expression is associated with metastatic progression and treatment history. (A and B) *PIMREG* and *BUB1* expression (log_2_ c.p.m.) across *SDHB* lesion types. The open triangles and squares denote *ATRX/TERT* alterations, respectively (Kruskal–Wallis *P* = 5.87 × 10^−9^ and 4.94 × 10^−9^). (C and D) Paired analysis of matched primary–metastatic tumors; the lines connect samples from the same patient. (E and F) Expression by prior treatment with DNA-damaging agents, such as chemotherapy or radiotherapy. (G and H) Kaplan–Meier analysis of metastasis-free survival stratified by *PIMREG* and *BUB1* expression.

PIMREG and BUB1 are mitotic regulators; PIMREG promotes the metaphase-to-anaphase transition, while BUB1 is a central component of the spindle assembly checkpoint that restrains this transition until all chromosomes achieve proper bi-orientation (28, 29, 30, 31). Beyond its canonical mitotic functions, PIMREG drives tumor aggressiveness through NF-κB activation and TWIST1 stabilization, promoting epithelial–mesenchymal transition (29). The highest PIMREG expression is observed in glioblastoma, where it contributes to temozolomide resistance (28). BUB1 is a master coordinator of kinetochore assembly and inhibits the anaphase-promoting complex (APC/C), thereby preventing premature sister chromatid separation (30). BUB1 exhibits a tumor suppressor paradox where partial loss-of-function promotes aneuploidy and tumorigenesis, while complete inactivation is lethal to cells, a vulnerability that may be therapeutically exploitable (30). The strong co-expression of PIMREG and BUB1 in metastatic PPGLs may reflect a coordinated transcriptional activation of the mitotic program, enabling sustained proliferation despite the metabolic stress inherent to SDH-deficient state. PIMREG and BUB1 may serve as prognostic biomarkers for metastatic potential in *SDHB*-related PPGLs. The dependence of metastatic PPGLs on mitotic checkpoint machinery may present a therapeutic opportunity for agents such as Aurora kinase A inhibitors and CDK4/6 (32, 33).

*BUB1* overexpression was validated independently by RT-qPCR in a cohort of 21 primary tumors and demonstrated functional dependence in hPheo1 cells, where genetic silencing of *BUB1* or pharmacological inhibition reduced viability, colony formation, migration, invasion, and induced apoptosis (34).

Genes co-expressed with *BUB1* form part of a larger mitotic network (Fig. 4A). The network is enriched for G2M checkpoint, E2F targets, glycolysis, and proliferation (Fig. 4B), and that includes known proliferation markers, such as Ki-67 (encoded by *MKI67* gene). Indeed, *BUB1* and *MKI67* are strongly correlated at the transcriptomic level (*R* = 0.952; Fig. 4C), although this correlation is not consistently reflected histologically (Fig. 4D). Unlike Ki-67, which is a marker rather than an effector and has been shown to be uncoupled from proliferation, BUB1 targeting (genetic and pharmacologic) resulted in halting of cellular proliferation and induction of apoptosis (34, 35, 36, 37).

**Figure 4:**
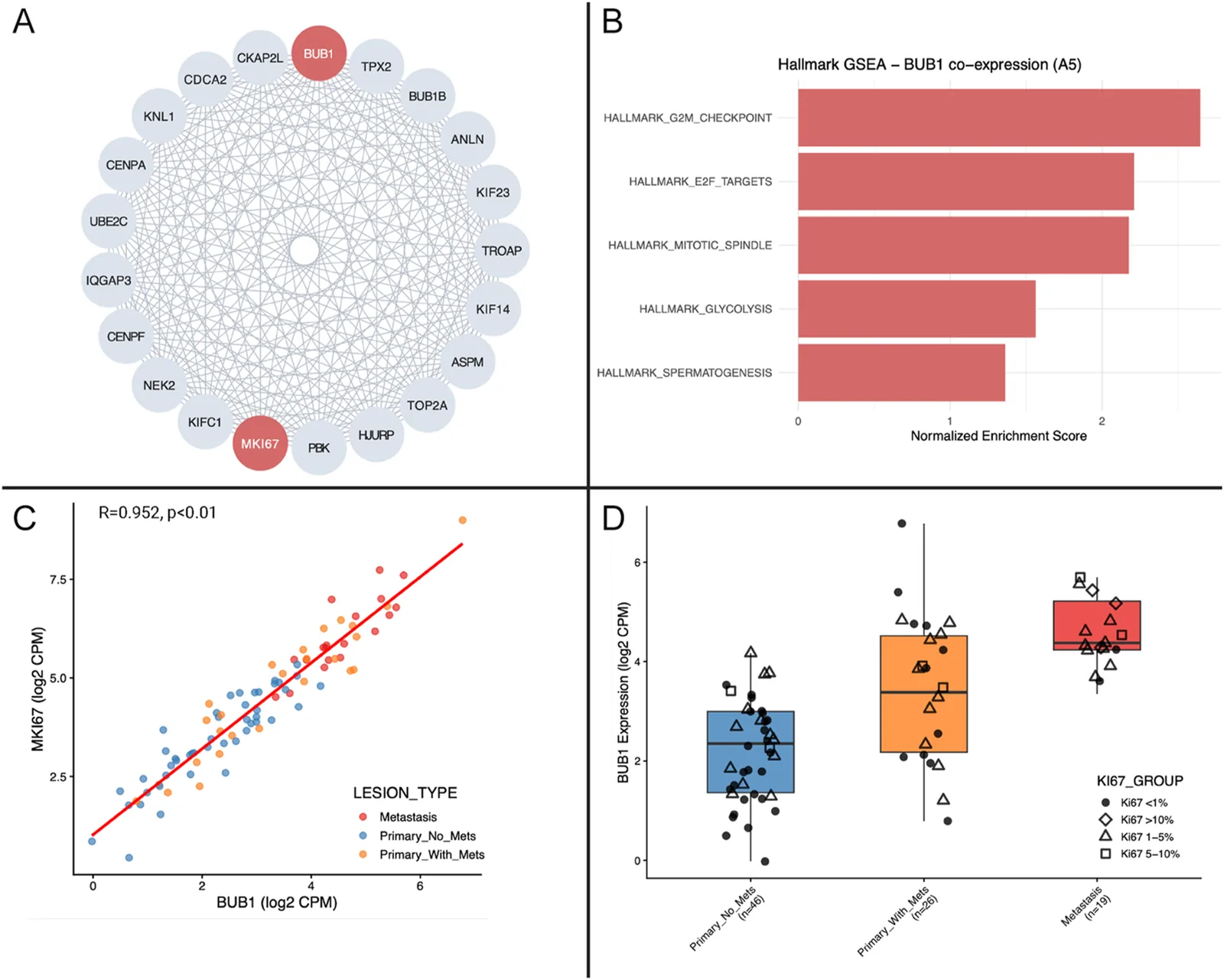
BUB1 co-expression mitotic gene network. (A) BUB1 co-expression network (top correlated genes from PPGLomics); *BUB1* and *MKI67* are highlighted (red). (B) Gene set enrichment analysis (GSEA) of the *BUB1* co-expression network (A5 cohort): Hallmark enrichment scores, showing enrichment for G2M checkpoint, E2F targets, mitotic spindle, and glycolysis. (C) Scatter plot of *BUB1* versus *MKI67* expression (log2 c.p.m.) across the A5 *SDHB* cohort, colored by lesion type. The two genes are strongly correlated (*R* = 0.952, *P* < 0.01). (D) *BUB1* expression by lesion category with tumors annotated by the histological Ki-67 index group (<1%, 1–5%, 5–10%, and >10%). BUB1 rises with metastatic status, yet several metastatic lesions show a comparatively low Ki-67 index despite high *BUB1* expression.

## Discussion

PPGLomics v1.0 addresses the unmet need for disease-specific transcriptomics analysis tools in PPGL research. While pan-cancer portals, such as cBioPortal and GEPIA, provide very valuable resources for common malignancies, their utility for rare cancers is limited by the absence of disease-relevant annotations. By focusing exclusively on PPGLs and incorporating detailed genotypic and phenotypic annotations, PPGLomics v1.0 enables analyses that would otherwise require substantial bioinformatics expertise. The current version of PPGLomics v1.0 focuses on transcriptomics data from two well-characterized cohorts. Future directions will expand the platform to incorporate additional multi-omics data, including proteomics, metabolomics, immunomics, and single-cell RNA sequencing data as these become available. By integrating transcriptomic data with disease-specific molecular, clinical, and other annotations, PPGLomics v1.0 provides a structured foundation for advanced downstream analyses. PPGLomics v1.0 is freely available at https://alkaissilab.shinyapps.io/PPGLomics/.

## Supplementary materials



## Declaration of interest

The authors declare that there is no conflict of interest that could be perceived as prejudicing the impartiality of the work reported.

## Funding

This work was supported in part by the Intramural Research Program of the *Eunice Kennedy Shriver* National Institute of Child Health and Human Development (NICHD) within the National Institutes of Health (NIH). The contributions of the NIH author(s) are considered Works of the United States Government. The findings and conclusions presented in this paper are those of the author(s) and do not necessarily reflect the views of the NIH or the US Department of Health and Human Services.

## Data availability

The PPGLomics Shiny application and associated code are publicly available at https://github.com/HussamAlkaissi/PPGLomics.

## References

[1] Lander ES, Linton LM, Birren B, et al. Initial sequencing and analysis of the human genome. Nature 2001 409 860–921. (10.1038/35057062) 11237011

[2] Berger B & Yu YW. Navigating bottlenecks and trade-offs in genomic data analysis. Nat Rev Genet 2023 24 235–250. (10.1038/s41576-022-00551-z) PMC10204111 36476810

[3] Wojcik MH, Larkin K, Cipicchio M, et al. Toward same-day genome sequencing in the critical care setting. N Engl J Med 2025 393 2063–2065. (10.1056/nejmc2512825) PMC12854144 41091060

[4] Weinstein JN, Collisson EA, Mills GB, et al. The cancer genome Atlas pan-cancer analysis project. Nat Genet 2013 45 1113–1120. (10.1038/ng.2764) PMC3919969 24071849

[5] Cerami E, Gao J, Dogrusoz U, et al. The cBio cancer genomics portal: an open platform for exploring multidimensional cancer genomics data. Cancer Discov 2012 2 401–404. (10.1158/2159-8290.cd-12-0095) PMC3956037 22588877

[6] Tang Z, Kang B, Li C, et al. GEPIA2: an enhanced web server for large-scale expression profiling and interactive analysis. Nucleic Acids Res 2019 47 W556–W560. (10.1093/nar/gkz430) PMC6602440 31114875

[7] Chandrashekar DS, Bashel B, Balasubramanya SAH, et al. UALCAN: a portal for facilitating tumor subgroup gene expression and survival analyses. Neoplasia 2017 19 649–658. (10.1016/j.neo.2017.05.002) PMC5516091 28732212

[8] Tang G, Cho M & Wang X. OncoDB: an interactive online database for analysis of gene expression and viral infection in cancer. Nucleic Acids Res 2022 50 D1334–D1339. (10.1093/nar/gkab970) PMC8728272 34718715

[9] Goldman MJ, Craft B, Hastie M, et al. Visualizing and interpreting cancer genomics data via the Xena platform. Nat Biotechnol 2020 38 675–678. (10.1038/s41587-020-0546-8) PMC7386072 32444850

[10] Huang J, Mao L, Lei Q, et al. Bioinformatics tools and resources for cancer and application. Chin Med J Engl 2024 137 2052–2064. (10.1097/cm9.0000000000003254) PMC11374212 39075637

[11] Richter S, Qiu B, Ghering M, et al. Head/neck paragangliomas: focus on tumor location, mutational status and plasma methoxytyramine. Endocr Relat Cancer 2022 29 213–224. (10.1530/erc-21-0359) PMC8942340 35171114

[12] Hamidi O, Young WF Jr., Iniguez-Ariza NM, et al. Malignant pheochromocytoma and paraganglioma: 272 patients over 55 years. J Clin Endocrinol Metab 2017 102 3296–3305. (10.1210/jc.2017-00992) PMC5587061 28605453

[13] Alkaissi H, Taieb D, Lin FI, et al. Approach to the patient with metastatic pheochromocytoma and paraganglioma. J Clin Endocrinol Metab 2025 110 2946–2963. (10.1210/clinem/dgaf259) PMC12448647 40317194

[14] Mete O, Asa SL, Gill AJ, et al. Overview of the 2022 WHO classification of paragangliomas and pheochromocytomas. Endocr Pathol 2022 33 90–114. (10.1007/s12022-022-09704-6) 35285002

[15] Pacak K. New biology of pheochromocytoma and paraganglioma. Endocr Pract 2022 28 1253–1269. (10.1016/j.eprac.2022.09.003) PMC9982632 36150627

[16] Fishbein L, Leshchiner I, Walter V, et al. Comprehensive molecular characterization of pheochromocytoma and paraganglioma. Cancer Cell 2017 31 181–193. (10.1016/j.ccell.2017.01.001) PMC5643159 28162975

[17] Dahia PL. Pheochromocytoma and paraganglioma pathogenesis: learning from genetic heterogeneity. Nat Rev Cancer 2014 14 108–119. (10.1038/nrc3648) 24442145

[18] Cascon A, Calsina B, Monteagudo M, et al. Genetic bases of pheochromocytoma and paraganglioma. J Mol Endocrinol 2023 70 e220167. (10.1530/jme-22-0167) 36520714

[19] Flynn A, Pattison AD, Balachander S, et al. Multi-omic analysis of SDHB-deficient pheochromocytomas and paragangliomas identifies metastasis and treatment-related molecular profiles. Nat Commun 2025 16 2632. (10.1038/s41467-025-57595-y) PMC11914184 40097403

[20] Lopez-Jimenez E, Gomez-Lopez G, Leandro-Garcia LJ, et al. Research resource: transcriptional profiling reveals different pseudohypoxic signatures in SDHB and VHL-related pheochromocytomas. Mol Endocrinol 2010 24 2382–2391. (10.1210/me.2010-0256) PMC5417372 20980436

[21] Qin Y, Yao L, King EE, et al. Germline mutations in TMEM127 confer susceptibility to pheochromocytoma. Nat Genet 2010 42 229–233. (10.1038/ng.533) PMC2998199 20154675

[22] Dahia PL, Ross KN, Wright ME, et al. A HIF1alpha regulatory loop links hypoxia and mitochondrial signals in pheochromocytomas. PLoS Genet 2005 1 72–80. (10.1371/journal.pgen.0010008) PMC1183527 16103922

[23] Shankavaram U, Fliedner SM, Elkahloun AG, et al. Genotype and tumor locus determine expression profile of pseudohypoxic pheochromocytomas and paragangliomas. Neoplasia 2013 15 435–437. (10.1593/neo.122132) PMC3612915 23555188

[24] Qin N, de Cubas AA, Garcia-Martin R, et al. Opposing effects of HIF1alpha and HIF2alpha on chromaffin cell phenotypic features and tumor cell proliferation: insights from MYC-associated factor X. Int J Cancer 2014 135 2054–2064. (10.1002/ijc.28868) 24676840

[25] Evenepoel L, van Nederveen FH, Oudijk L, et al. Expression of contactin 4 is associated with malignant behavior in pheochromocytomas and paragangliomas. J Clin Endocrinol Metab 2018 103 46–55. (10.1210/jc.2017-01314) 28938490

[26] Burnichon N, Vescovo L, Amar L, et al. Integrative genomic analysis reveals somatic mutations in pheochromocytoma and paraganglioma. Hum Mol Genet 2011 20 3974–3985. (10.1093/hmg/ddr324) 21784903

[27] Backman S, Botling J, Nord H, et al. The evolutionary history of metastatic pancreatic neuroendocrine tumours reveals a therapy driven route to high-grade transformation. J Pathol 2024 264 357–370. (10.1002/path.6348) 39360347

[28] Serafim RB, Cardoso C, Arfelli VC, et al. PIMREG expression level predicts glioblastoma patient survival and affects temozolomide resistance and DNA damage response. Biochim Biophys Acta Mol Basis Dis 2022 1868 166382. (10.1016/j.bbadis.2022.166382) 35301087

[29] Jiang L, Ren L, Zhang X, et al. Overexpression of PIMREG promotes breast cancer aggressiveness via constitutive activation of NF-kappaB signaling. EBioMedicine 2019 43 188–200. (10.1016/j.ebiom.2019.04.001) PMC6557765 30979686

[30] Bolanos-Garcia VM & Blundell TL. BUB1 and BUBR1: multifaceted kinases of the cell cycle. Trends Biochem Sci 2011 36 141–150. (10.1016/j.tibs.2010.08.004) PMC3061984 20888775

[31] Malumbres M. Physiological relevance of cell cycle kinases. Physiol Rev 2011 91 973–1007. (10.1152/physrev.00025.2010) 21742793

[32] Ciciro Y, Ragusa D & Sala A. Expression of the checkpoint kinase BUB1 is a predictor of response to cancer therapies. Sci Rep 2024 14 4461. (10.1038/s41598-024-55080-y) PMC10891059 38396175

[33] Zheng D, Li J, Yan H, et al. Emerging roles of Aurora-A kinase in cancer therapy resistance. Acta Pharm Sin B 2023 13 2826–2843. (10.1016/j.apsb.2023.03.013) PMC10372834 37521867

[34] Ahmadi M, Zargar SJ, Alkaissi H, et al. BUB1 as a candidate non-oncogene addiction vulnerability in metastatic phaeochromocytoma/paraganglioma. Sci Rep 2026 16 18544. (10.1038/s41598-026-49408-z) PMC13269497 42014906

[35] Sobecki M, Mrouj K, Camasses A, et al. The cell proliferation antigen Ki-67 organises heterochromatin. eLife 2016 5 e13722. (10.7554/elife.13722) PMC4841783 26949251

[36] Cuylen S, Blaukopf C, Politi AZ, et al. Ki-67 acts as a biological surfactant to disperse mitotic chromosomes. Nature 2016 535 308–312. (10.1038/nature18610) PMC4947524 27362226

[37] Cidado J, Wong HY, Rosen DM, et al. Ki-67 is required for maintenance of cancer stem cells but not cell proliferation. Oncotarget 2016 7 6281–6293. (10.18632/oncotarget.7057) PMC4868756 26823390

