# Supplementary material for: PPGLomics: an interactive platform for pheochromocytoma and paraganglioma transcriptomics
Source: Endocr Relat Cancer. 2026 Sep 22;33(9):e260140. doi: 10.1530/ERC-26-0140 (PMC13641061; doi:10.1530/ERC-26-0140)
Supplement: Supplementary file 1 [file ERC-26-0140_supplementary_figure_1.pdf]

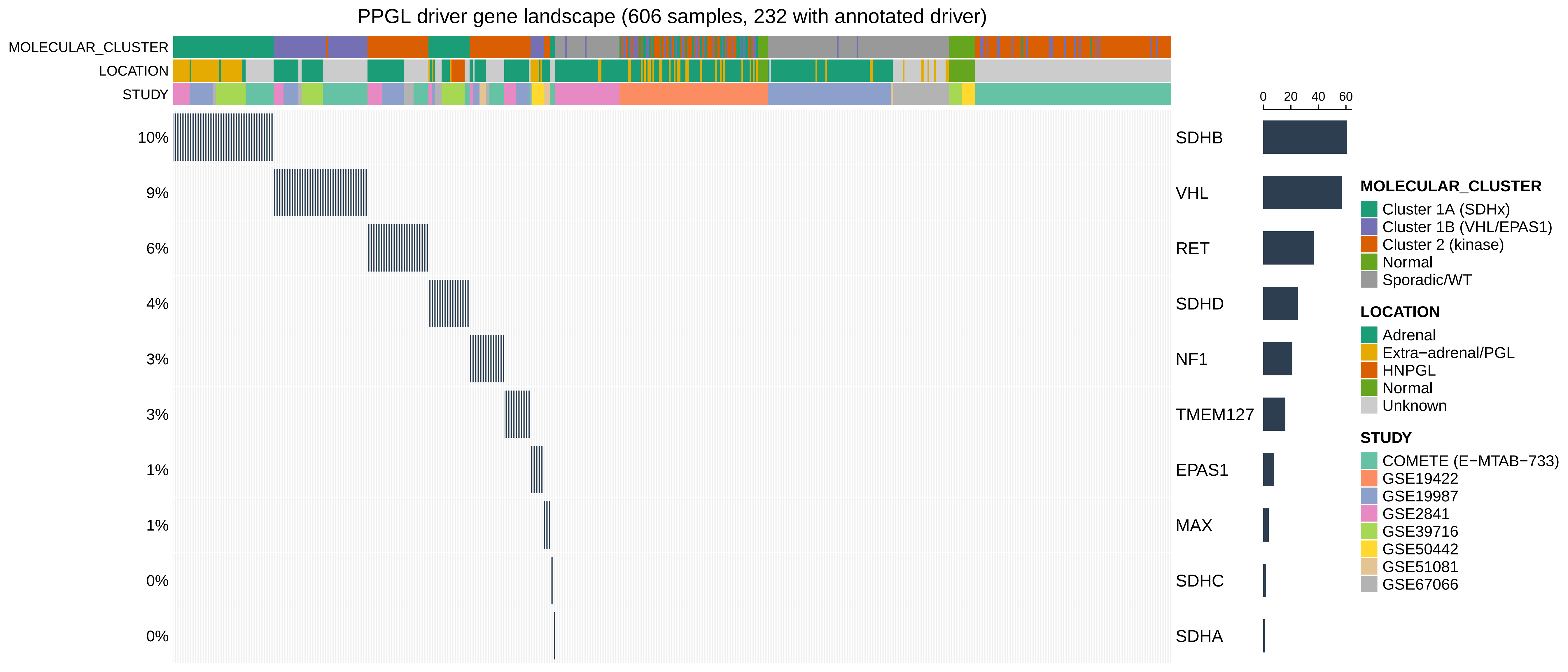
PPGL driver gene landscape (606 samples, 232 with annotated driver)
MOLECULAR_CLUSTER
LOCATION
STUDY
10%
9%
6%
4%
3%
3%
1%
1%
0%
0%
SDHB
VHL
RET
SDHD
NF1
TMEM127
EPAS1
MAX
SDHC
SDHA
0
20
40
60
MOLECULAR_CLUSTER
Cluster 1A (SDHx)
Cluster 1B (VHL/EPAS1)
Cluster 2 (kinase)
Normal
Sporadic/WT
LOCATION
Adrenal
Extra−adrenal/PGL
HNPGL
Normal
Unknown
STUDY
COMETE (E−MTAB−733)
GSE19422
GSE19987
GSE2841
GSE39716
GSE50442
GSE51081
GSE67066
